# Subjective expectations for future and mortality among middle-aged and older adults

**DOI:** 10.1097/MD.0000000000019421

**Published:** 2020-04-24

**Authors:** Jae Woo Choi, Jae-Hyun Kim, Ki Bong Yoo

**Affiliations:** a College of Pharmacy, Yonsei Institute of Pharmaceutical Sciences, Yonsei University, Incheon; b Department of Health Administration, College of Health Science, Dankook University, Cheonan, South Korea; c Department of Health Administration, Department of Information & Statistics, Yonsei University, Wonju, Korea.

**Keywords:** longitudinal study, mortality, older adults, subjective expectations for future

## Abstract

The purpose of this study is to categorize various elements for the expectations for the future using factor analysis and identify association between categories of the subjective expectations for the future and mortality among middle-aged and older adults.

Data from the Korean Longitudinal Study of Aging from 2006 to 2016 was assessed using longitudinal data analysis and 9,844 research subjects were included at baseline in 2006. Our modeling approach was based on Cox proportional hazards models for mortality.

We indicated 3 categories (individual factor, national factor, and combined factor) of 12 subjective expectations for the future using factor analysis. The negative expectations for the future of all factors [individual factor: hazard ratio (HR), 1.65, 95% confidence interval (CI), 1.41–1.93; national factor: HR, 1.20, 95% CI, 1.06–1.37; combined factor: HR, 1.16; 95% CI, 1.02–1.32] were more likely to have an increased risk of all-cause mortality than those in the positive expectations for the future. Older adults were more likely to be affected by negative expectations for the future in national factor compared to middle-aged adults (HR, 1.22; 95% CI, 1.05–1.41).

Increasing positive expectations for the future is an important consideration for improvement in health. Policy makers need to consider that changes of national policy would affect health in older adults.

## 1 Introduction

Subjective expectations for the future play a central role in individual decision-making.^[1]^ When subjective expectations are inaccurate (eg, too pessimistic) this may result in non-optimal decisions.^[2]^ In addition, negative expectations for the future and ambivalent expectations can be associated with low wellbeing and hopelessness.^[3]^ Hopelessness about the future is central in cognitive accounts of depression^[4]^ and subjective expectations for the future also may be associated with physical health.

Representative theme on subjective expectations for the future is subjective life expectancy, which is a concept that assesses individuals’ expectations about their time horizon.^[5,6]^ Subjective life expectancy may just be a reflection of an optimistic (or pessimistic) life orientation.^[7]^ In this view, subjective life expectancy is linked with mortality through direct or indirect influences of emotions or dispositions on physiological state and health. Previous studies on subjective expectancy for the future have been conducted to assess the predictive value of subjective life expectancy on actual mortality.^[8–11]^

Previous research also found that subjective judgment in regards to economic circumstances, health conditions, and well-being, which is also a type of subjective expectancy for the future, is associated with mortality.^[12–14]^ Additionally, an individual has expectations for the future in various dimensions as well as in personal aspect. A nation is responsible for ensuring the right to health among citizens and people expect to be supported by government in their essential needs.^[15]^ Declining birth rates and increased participation of women in the labor market in Korea have greatly reduced expectations for care from family members among middle aged and older adults.^[16]^ Recently, changes in attitude toward support for parents have been noticeable. The proportion of citizens responding that families should support their parents decreased from 89.9% in 1998 to 31.7% in 2014.^[17]^ These social changes are factors that increase expectancy for the nation. However, Research on mortality by subjective expectancy for the future according to diverse dimensions has been limited to middle-aged and older adults.

Therefore, this study aims to categorize many elements of the expectations for the future using factor analysis and identify association between categories of the subjective expectations for the future and mortality among middle-aged and older adults using nationwide representative longitudinal data. This study also estimates the age-specific effect of the subjective expectancies for future on deaths while adjusting covariates.

## 2 Methods

### 2.1 Study sample and design

Data were obtained from the 2006, 2008, 2010, 2012, 2014, and 2016 waves of the Korean Longitudinal Study of Aging. Korean Longitudinal Study of Aging conducted a multistage stratified cluster sampling based on 15 geographical areas and housing types across the nation to create nationally representative longitudinal data of Koreans aged 45 years or more by the Korea Labor Institute. In the first baseline survey in 2006, 10,254 individuals in 6171 households (1.7 per household) were interviewed using the Computer-Assisted Personal Interviewing method. The second survey, in 2008, followed up with 8688 subjects, who represented 84.7% of the original panel. The third survey, in 2010, followed up with 7920 subjects, who represented 77.2% of the original panel, the fourth survey, in 2012, followed up with 7486 subjects, who represented 73.0% of the original panel, the fifth survey, in 2014, followed up with 7029 subjects, who represented 68.6% of the original panel, and the sixth survey, in 2016, followed up with 6618 subjects, who represented 64.5% of the original panel. To estimate the association between subjective expectancy for the future and mortality among people 45 years or older, we included 9844 participants at baseline 2006 with no missing information.

### 2.2 Independent variable

Survey on subjective expectancy for the future consists of 12 items in our database. The items measure a continuum of subjective probabilities by obtaining responses to the following components:

(1) legacy gift (when I think about all the assets that I possess now, I can leave more than about 90,000 dollars),
(2) life expectancy {what is the percent chance that you will live to be [75 (if age is 64 or less)/80 (if age is 65–69)/85 (if age is 70–74)/90 (if age is 75–79)/95 (if age is 80–84)/100 (if age is 85–94)]/105 (if age is 95–99)/110 (if age is 100 or more)]? the target age in expectation is determined by respondents’ current age},
(3) the standard of living in the future (I am going to lower my standard of living in the future),
(4) socio-economic level of their children (I think that the younger generation can live in a better economic/social environment than our generation),
(5) security for the aged (I can guarantee my old age in the country),
(6) unification of North and South Korea (in the next 10 years, the 2 Koreas are likely to be unified),
(7) recession (in the next 10 years, Korea's economy is likely to suffer a severe recession),
(8) stable real estate market (the Korean real estate market is likely to stabilize in the next 10 years),
(9) health status (how satisfied are you with your health condition?),
(10) the state of one's finances (how satisfied are you with your economic situation?), (11) family relationship (How satisfied are you with your relationship with your spouse or children?), and
(11) relative quality of life (Compared to your peers, how satisfied are you about your overall quality of life (happiness)?).

We conducted an exploratory factor analysis (principal component analysis with varimax rotation which is a statistical technique used at one level of factor analysis) for subjective expectations for future. The factor analysis analyzes the correlations between item and variables to identify factors with high correlation. The factor analysis indicated 3 categories of 12 items, which measure subjective expectancy for the future.

### 2.3 Dependent variable

All-cause mortality during the time interval from year 2006 to the end of follow-up was the main outcome of the study. Death over a maximum follow-up period of 10 years was determined by death certificates.

### 2.4 Covariates

Covariates were collected: age (45–54, 55–64, 65–74 and ≥65 years), gender, residential region (urban and rural), education (elementary, middle, high school and ≥college), marital status [married and single (including separated, divorced)], employment status (yes and no), health security (national health insurance and medical aid), smoking (non-smoker, former smoker and smoker), alcohol consumption (never, former drinker and drinker), and comorbidities of hypertension, diabetes, cancer, chronic obstructive pulmonary disease, liver disease, heart disease, cerebrovascular diseases, mental illness and arthritis or rheumatoid arthritis (0, 1, 2, and ≥3).

### 2.5 Statistical analysis

Chi-square test, log-rank test, factor analysis, and Cox proportional hazards models were used to investigate the association between subjective expectancy for the future and mortality. Adjusted hazard ratio (HR) was calculated by cox proportional hazard model. Covariates of interest from all subjects were added to the model to determine their effects on the probability of reporting mortality. The outcome variable was survival time, which was measured from the date of enrollment to death or censoring (up to 8 years). For all analyses, the criterion for statistical significance was *P* ≤.05, 2-tailed. All analyses were conducted using the SAS statistical software package, version 9.4 (SAS Institute Inc., Cary, NC, USA). This study was exempt from ethical approval from our institution's IRB.

## 3 Results

### 3.1 Sample characteristics

Table 1 shows the baseline characteristics of participants. As shown in Table 1, of the 9,844 individuals at baseline in 2006, middle-aged (45–64) and older (≥65) adults were 5,845 (59.3%), 3,999 (40.7%) and about 10.2%, 65.3% of each group died during the follow-up period, respectively. 56.6% were female and 46.9% had elementary or lower educational attainment. 64.6% lived in urban and about 2 third were married. 38.9% were workers of employment status and 5.5% were those who enrolled in medical aid, which is a public medical assistance program targeted at poor individuals. About 18.9% were present smokers and 55.4% were present drinkers, and half of participants had chronic diseases.

**Table 1:**
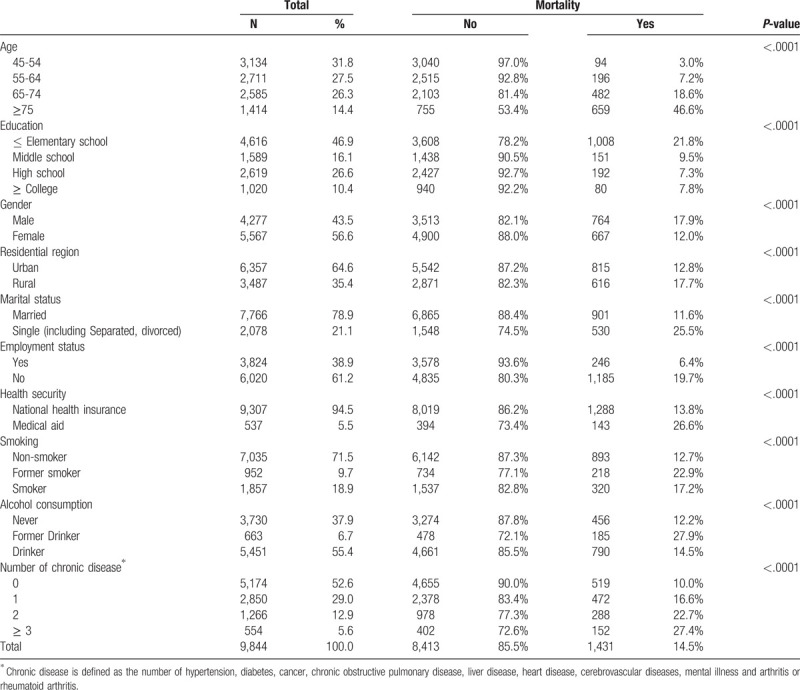
General characteristics of subjects included for analysis.

### 3.2 Factor analysis

Table 2 indicates factor analysis for categorizing a few elements that consist of expectations for the future. We categorized 3 groups [individual factor, national factor, and combined factor (individual and nation)] according to scores by factor analysis, and the group as well as the score are as follows:

**Table 2:**
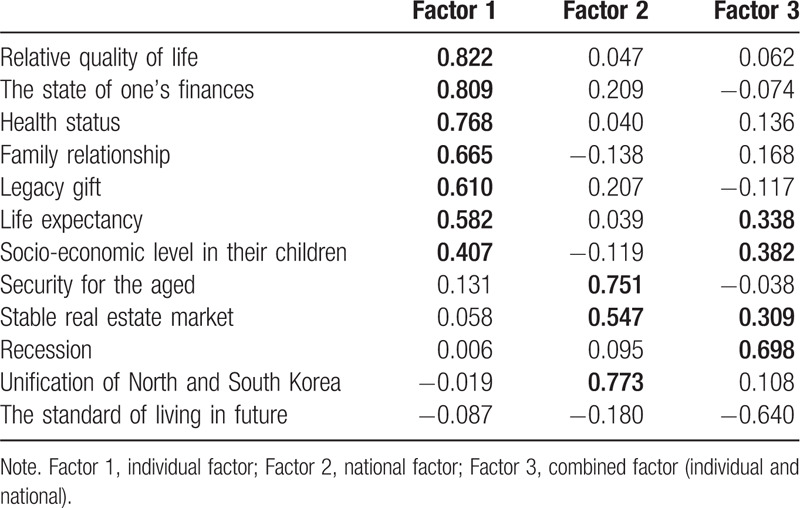
Results of Factor analysis.

(i) factor 1 (individual factor): relative quality of life (0.822), the state of one's finances (0.809), health status (0.768), family relationship (0.665), legacy gift (0.610), life expectancy (0.582), and socio-economic level in their children (0.407); (ii) factor 2 (national factor): unification of North and South Korea (0.773), security for the aged (0.751), and stable real estate market (0.547); (iii) factor 3 (combined factor): recession (0.698), socio-economic level in their children (0.382), life expectancy (0.338), and stable real estate market (0.309).

### 3.3 Relationship between expectations for the future and mortality

**Figure 1:**
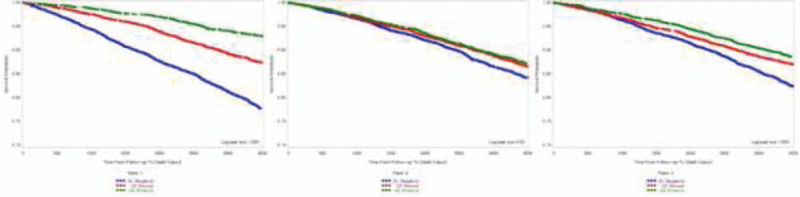
Kaplan-Meier curve for all-cause mortality according to factors.

Figure 1 shows the Kaplan-Meier curve for all-cause mortality according to factors. All of log-rank tests were statistically significant. In the fully adjusted model (Table 3), each factor was associated with all-cause mortality. In factor 1 (individual factor), Q1 (negative expectations for future) had a higher risk for all-cause mortality compared to Q3 (positive expectations for future) [HR, 1.65; 95% confidence interval (CI), 1.41–1.93]. Those in Q1 of factor 2 (nation factor) were more likely to have an increased risk of all-cause mortality than those in Q3 (HR, 1.20; 95% CI, 1.06–1.37). Those in Q1 of factor 3 (combined factor) had a higher risk for all-cause mortality compared to Q3 (HR, 1.16; 95% CI, 1.02–1.32). Males presented significantly high HR compared to females (HR, 2.43; 95% CI, 2.08–2.84), and a clear upward trend in all-cause mortality was observed with increasing age. Those with elementary or lower educational attainment were more likely to have an increased risk of all-cause mortality than those with college or above (HR, 1.38; 95% CI, 1.08–1.76), and those who were single (including separated, divorce) presented significantly higher HR than those who were married (HR, 1.42; 95% CI, 1.24–1.62). Non-smokers and drinkers presented significantly higher HR than smokers (HR, 1.32; 95% CI, 1.11–1.58) and non-drinkers (HR, 1.16; 95% CI, 1.01–1.33), respectively. People with a job were more likely to have a decreased risk of all-cause mortality than those without a job (HR, 0.59; 95% CI, 0.50–0.69), and a clear upward trend in all-cause mortality was observed with increasing number of chronic diseases.

**Table 3:**
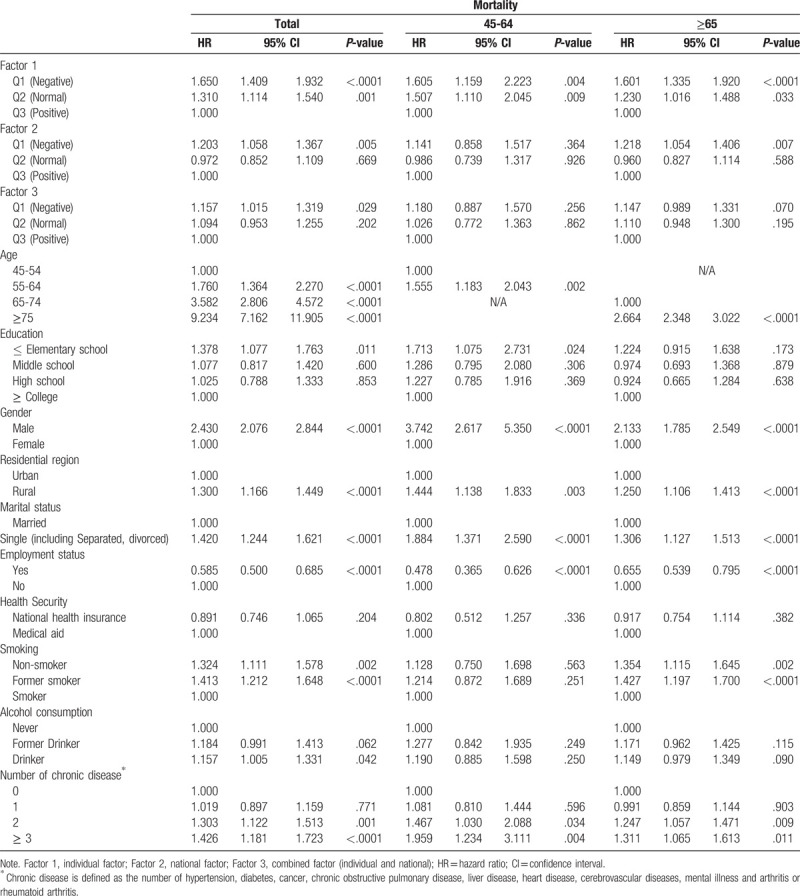
Factors associated with suicide mortality in middle-aged and older adults.

### 3.4 Age specific relationship between expectations for the future and mortality

Table 3 shows age specific relationship between expectations for the future and mortality. In the middle aged group (45–64), study subjects with 2 (HR, 1.47; 95% CI, 1.03–2.09) or more chronic diseases (HR, 1.96; 95% CI, 1.23–3.11) were more likely to have an increased risk of all-cause mortality compared to those without chronic diseases, respectively. Q1 (negative expectations for future) of factor 1 (individual factor) had a higher risk for all-cause mortality compared to Q3 (positive expectations for future) (HR, 1.65; 95% CI, 1.41–1.93). In older adults, study subjects with 2 (HR, 1.25; 95% CI, 1.06–1.47) or more chronic diseases (HR, 1.31; 95% CI, 1.07–1.61) were more likely to have an increased risk of all-cause mortality compared to those without chronic diseases, respectively. Q1 (negative expectations for future) of factor 1 (individual factor) and factor 2 (national factor) were more likely to have an increased risk of all-cause mortality than Q3 (positive expectations for future) (HR, 1.60, 1.22; 95% CI, 1.34–1.92, 1.05–1.41), respectively. There was no statistical difference in factor 3 (combined factor) in age specific analysis results.

## 4 Discussions

The primary purpose of this study was to examine the association between subjective expectations for the future and mortality in middle-aged and old adults in South Korea over 10-year study follow-up, from which there were 3 main findings. First, we indicated 3 categories (individual factor, national factor, and combined factor) of 12 subjective expectations for the future using factor analysis. Second, negative expectations for future of all factors were more likely to have an increased risk of mortality than positive expectations for future. Third, negative expectations for future of nation factor were more likely to have an increased risk of all-cause mortality than positive expectations for future only among older adults, and not middle-aged adults.

Specific (psychological) construct that has been most consistently associated with various health outcomes in dispositional optimism (as measured by the Life Orientation Test) and conceived as largely stable trait (one could read this as resistant to change or any kind of intervention).^[18–20]^ On the other hand, there are other, more potentially modifiable constructs, such as positive expectancies and state optimism – that have been also associated with various outcomes. Our findings showed that negative expectations for future of all factors were associated to have an increased risk of mortality and we suggest having positive expectations for future is one of the important factors for health.

Subjective expectations about the future are a central component of human cognition. It involves the ability to project the self forward in time in order to pre-experience an event.^[21]^ Previous studies have demonstrated the relationship between expectancy for future and well-being. For instance, previous research has shown that patterns of positive future-directed thinking are linked to reported higher life satisfaction and quality of life, more adaptive behaviors, less distress, and overall higher well-being.^[22–24]^ By contrast, negative expectations for the future are associated with and substance abuse, maladaptive behaviors, such as alcohol, more avoidance coping, less persistence, facing life's challenges, and poor health.^[25,26]^ Moreover, reduced anticipation of future positive events is a defining characteristic of depression, whereas anxiety is characterized by an increase in the number of perceived negative future events.^[27–29]^ These previous studies support our main findings that negative expectations for the future were more likely to have an increased risk of mortality than those in positive expectations for the future.

Regarding age-specific association with expectations for the future and mortality, this study also suggests that negative expectations for the future of national factor (unification of North and South Korea, security for the aged, and stable real estate market) were more likely to have an increased risk of mortality than those having positive expectations for future among older adults. The results need to be interrupted by Korean social perspectives. Senior citizens usually retire from their jobs and receive financial support by the government or their children. However, the proportion of assistance resource has recently changed in South Korea. According to 2018 the aged statistics, cost of living from children of older adults decreased from 39.2% in 2011 to 25.7% in 2017.^[30]^ However, cost of living from the government and social organization increased from 9.1% 2011 to 12.5 in 2017. This means that older adults are more sensitive to government policy compared to other age groups. Also, Korean older adults have experienced the Korean War that occurred with the invasion of North Korea in 1950 and their family were scattered by the war. Therefore, most of older adults want unifications more compared to other generations. These features result in increasing expectations and concerns for the future in national aspects among older adults. In other words, older adults may be affected by negative expectations for the future in nation factor compared to middle-aged adults.

Several potential limitations of the present study should be noted. First, data were gathered from self-reports of socio-demographic factors and health risk factors, and self-report is an imperfect indicator of actual behavior. Second, we did not assess other factors (eg, family medical history) that may account for the association between subjective expectations for future and mortality. Third, present study only examined the effects of subjective expectations on all-cause mortality but not specific-cause mortality because our data did not include information for specific cause mortality. Each cause of death is given an equal weight, even though its individual contribution may not have been equally important – the relative importance of each cause of death is not considered.

Despite these limitations, this study has various strengths, particularly with its use of a nationwide representative sample and the 10-year follow-up database. We also prospectively analyzed a large number of individuals from longitudinal data of a well-defined and comprehensively studied sample of middle-aged and older adults to examine the association between subjective expectations for the future and mortality. Therefore, with the rapidly ageing population in Korea, subjective expectations for the future are a reasonably good predictor of future mortality. Our findings leave little room for doubt about whether subjective expectancy for the future should be taken seriously to understand future survival and to meaningfully intervene in preventing early mortality.

Inequality in mortality due to varying socioeconomic status has been shown in different countries.^[31,32]^ People with poor socioeconomic status and educational attainment may have lower expectations for the future, and this low expectancy could lead to the increase in mortality. Therefore, further research needs to examine subjective expectations for the future due to different socio-demographic factors more accurately, as well as how socio-demographic and subjective expectations for future factors affect mortality.

## 5 Conclusions

This study found that negative expectations for future were likely to have an increased risk of mortality than those in the positive expectations for future. Older adults are more likely to be affected negative expectations for future in national aspect compared to middle-aged adults. Increasing positive expectations for future is important consideration for improvement of health. Policy makers need to consider that changes of nation policy would affect health in older adults.

## Acknowledgment

The authors would like to thank an experienced editor for the English language editing.

## Author contributions

Kim JH designed the study and analyzed the data. Choi JW searched the previous literatures and wrote the draft. Ki Bong Yoo contributed to conception, design, revision, and final approval. All authors have approved the final manuscript.
